# Optimum Processing Conditions for Flavor-Enhancing Green Laver Chips Using Reaction Flavor Technology

**DOI:** 10.3390/foods13233876

**Published:** 2024-11-29

**Authors:** Jeong-Min Heo, Changheon Lee, Yong-Jun Cha, Daeung Yu

**Affiliations:** 1 Department of Food and Nutrition, Changwon National University, Changwon 51140, Republic of Korea; wjdals3471@naver.com (J.-M.H.); yjcha@changwon.ac.kr (Y.-J.C.); 2 Interdisciplinary Program in Senior Human Ecology, Changwon National University, Changwon 51140, Republic of Korea; ckdgjs3306@changwon.ac.kr

**Keywords:** green laver, reaction flavor, air-frying, flavor-enhancing, chip, pyrazine

## Abstract

The optimum processing conditions for green laver chips were determined using response surface methodology (RSM) to improve taste and reduce off-flavors by applying reaction flavor and air-frying techniques. The optimum composition (*w*/*w*) for the chips included 20% green laver, 20% hairtail surimi, and 60% flour. Additional ingredients included distilled water (90 mL) with GDL (3 g), NaHCO₃ (2 g), salt (1 g), sugar (12 g), roasted soybean powder (1.5 g), and reaction flavor solution (RFS, 10 mL). The mixture was kneaded, shaped, dried at 50 °C for 2 h, and air-fried at 195 °C for 80 sec. The resulting green laver chips showed overall acceptance and brittleness values of 7.00 ± 0.74 and 5.89 ± 0.59 N, respectively, with absolute residual errors of 8.43% and 7.07%. The optimum reaction flavor precursors for green laver chips were determined to be threonine (1.0 g%), proline (1.0 g%), glycine (1.4 g%), methionine (0.05 g%), and glucose (2 g%). Flavor analysis revealed that green laver chips with reaction flavor (GLCR) contained 13 alkylpyrazines with corn-like and nutty odors, and 2-acetylpyrrole, which contributed a popcorn-like odor. In contrast, green laver chips without reaction flavor (GLC) predominantly contained straight-chain aldehydes with undesirable odors. The heating process in the air fryer appeared to reduce the aldehyde content and promote pyrazine formation, significantly enhancing GLCR’s flavor.

## 1. Introduction

Seaweed, a valuable resource, is found in various environments and under extreme conditions. It is rich in complex organic compounds such as chlorophyll, xanthophylls, carotenoids, secondary metabolites, and primary metabolites [1]. Notably, it contains sulfated branched heteropolysaccharides, such as phlorotannins and fucoidan, which are rarely found in terrestrial plants and are strongly linked to a reduced risk of chronic disorders such as cancer, hyperlipidemia, and coronary heart disease [2]. The abundant water-soluble dietary fiber in seaweed effectively helps manage diabetes by enhancing blood sugar control and improving lipid metabolism [3]. These unique properties have led to the increasing use of seaweed as additives in the production of functional foods, thereby increasing its value and recognition as a health food [4,5]. This trend underscores the growing importance of seaweed in the food industry and its potential for further research.

There are about 750 kinds of seaweed in Korea, but it is known that only about 30 kinds are used for food [6]. Among them, green laver (*Enteromorpha prolifera*), belonging to the Ulvaceae family of the Ulvales order, is produced on the west and south coasts of Korea [7]. In 2023, 8287 M/T of green lavers were produced, a 62.9% increase from 2022 (5088 M/T) [8]. Koreans often use green laver for salads and soups. Dried green laver contains 9 to 14% crude protein, 32 to 36% crude ash, and ω-3 and ω-6 polyunsaturated fatty acids at 10.4 and 10.9 g/100 g, respectively [9]. In addition, Jung et al. [10] reported that the high amount of amino acids, including glutamic acid and aspartic acid, contributes to the umami taste of green laver. Koo et al. [11] reported that the total dietary fiber of green laver accounts for 29.8% (dry basis), and these dietary fibers consist of glucuronorhamnoxyloglycan containing sulfate (20%). Furthermore, it has been reported that green laver is rich in sulfuric acid polysaccharides with anticoagulant, anti-allergic, antiviral, and anti-cancer properties [12,13]. Additionally, it contains significant amounts of phycocyanin and carotenoid pigments, which are associated with anti-inflammatory, antioxidant, and neuroprotective properties [14].

Unlike laver, there have been relatively few studies on the food composition of green laver because it is not a popular food ingredient in Korea [15,16]. Recently, studies on its food processability, such as in raw noodles [17], butter cookies [18], morning bread [6], and ganache [19], have been conducted. However, these studies remain fewer in comparison to those focused on laver-based processed products.

This study applied air-frying technology to examine the processability of making green laver chips with excellent health functionality. Air-frying technology circulates air (up to 200 °C) generated by the internal heating element to the internal fan, heating it via convection. This method has been reported to have the advantage of suppressing quality degradation due to lipid rancidity during storage compared to the traditional deep-frying method used for most seaweed-based snacks [20]. Products such as fish snacks [21], seaweed snacks [20,22], and sweet potato snacks [23], which have a lower fat content due to the application of air-frying technologies, have also been reported.

On the other hand, green laver presents many challenges in processability because consumers have mixed opinions about its grassy odor and the seaweed’s characteristic fishy smell [24]. Yi and Yoon [25] reported that the more green laver is added during pound cake processing, the stronger the odor, negatively affecting overall preference.

Given the unique grassy odor and seaweed’s fishy smell, which consumers either like or dislike, there is a clear need to improve the sensory quality of green laver. This study aims to address this need by applying reaction flavor technology, a promising approach to enhancing the sensory quality of green laver products. Reaction flavor technology is produced through the Maillard reaction, where amino acids and sugar components serve as precursors [26]. It has been widely used to induce and generate positive savory and characteristic flavor components in the cooking and processing of food for a long time [27]. The production of natural crab flavor from red crab by-products [28], the development of beef flavor from hydrolyzed vegetable protein (HVP) [29], and the creation of savory flavor from soy sauce residue hydrolysates [26] have been reported.

Therefore, this study established the optimum processing conditions identified through the application of response surface methodology to process green laver chips with green laver as the main component. By reducing the product’s odor through the application of reaction flavor and air-frying technology, the taste and flavor were enhanced, resulting in a processed green laver chip with improved lipid oxidation stability.

## 2. Materials and Methods

### 2.1. Materials

The dried and pulverized raw material for green laver chips (*Enteromorpha prolifera*) was purchased from Food-ai (Hanam, Republic of Korea), filtered through an 80-mesh sieve (Supply Filter Tech Co., Ansan, Republic of Korea), stored frozen at −24 °C, and used in the experiment in a specified amount. Frozen hairtail surimi (Xiangshan Shipu Goulog Aquatic Products Co., Ningbo, China) was purchased from GNK (GNK Co., Busan, Republic of Korea), divided into approximately 50 g, packed in a zip-lock bag (LDPE, 25 cm × 30 cm, Cleanwrap Co., Seoul, Republic of Korea), and stored at −24 °C for the experiment. β-Gluconolactone (GDL, manufactured at USA) was purchased from ESfood Co. (Gunpo, Republic of Korea) and sodium bicarbonate (NaHCO₃, France product) was purchased from Bread Garden Co. (Seongnam, Republic of Korea). As minor ingredients, sugar (CJ Cheiljedang Co., Incheon, Republic of Korea), multi-purpose flour (referred to as flour below) (CJ Cheiljedang Co., Incheon, Republic of Korea, originated from USA and Australia), roasted soybean powder (Cheong Eun F&B Co., Goyang, Republic of Korea), and table salt (CJ Cheiljedang Co., Busan, Republic of Korea) were purchased at a local market in Changwon, Republic of Korea. Additionally, amino acids (methionine, proline, glycine, and threonine) and glucose were applied to reaction flavor technology with free support from Vixol (Ansan, Republic of Korea).

### 2.2. Selection and Preparation of Optimum Reaction Flavor Solution (RFS)

Based on the methods of Cha and Wang [26] and Cha [30], the reaction flavor solution (RFS), which was added to impart a positive effect on the flavor of the green laver chip product, was prepared as follows. Four amino acids, including threonine (1.00 g), proline (1.00 g), glycine (1.40 g), and methionine (0.05 g), along with glucose (2.00 g), were selected according to the optimum mixing ratio through a ranking test based on preliminary experiments with green laver chips, and were combined with 100 mL of distilled water to dissolve the mixture. The solution was subsequently reacted in an autoclave (LAC-5080SD Co., Ltd., Namyangju, Republic of Korea) at 98 °C for 90 min. The RFS was subsequently integrated into the processing procedure for green laver chips.

### 2.3. Preparation of Green Laver Chips

**Figure 1:**
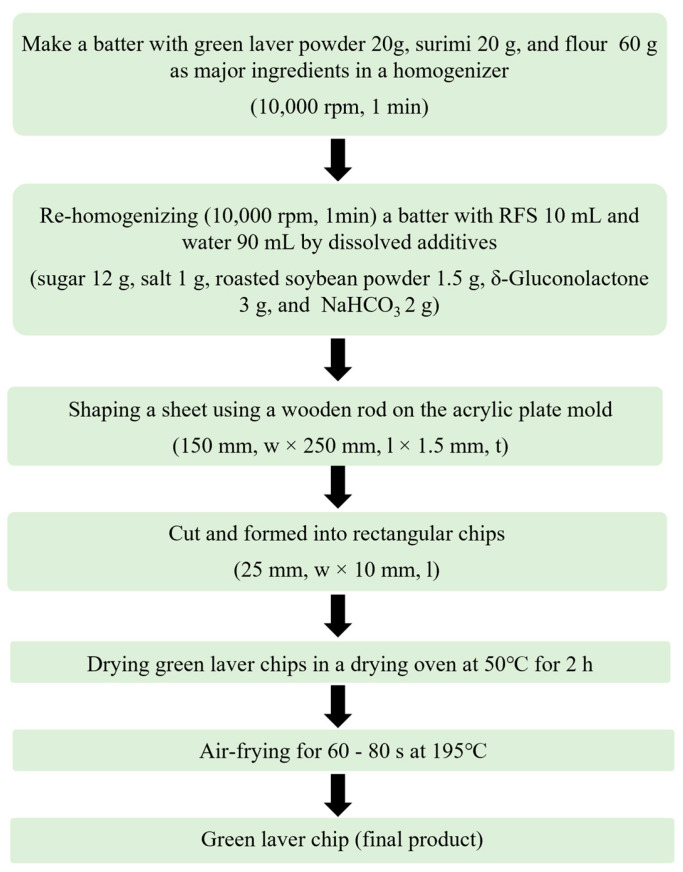
Flow chart for the preparation of green laver chips.

**Figure 2:**
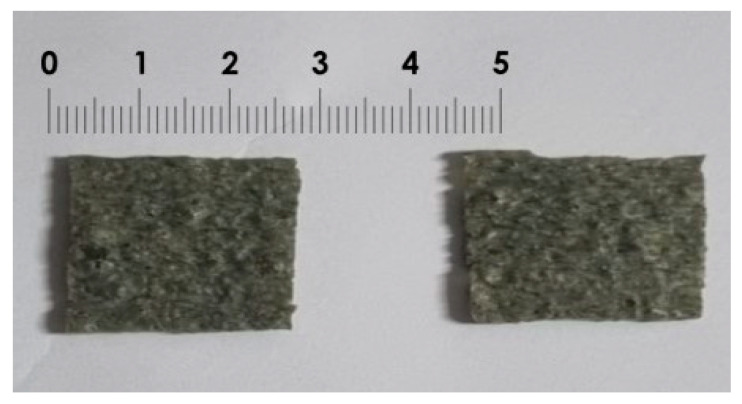
Green laver chip product.

The processing of the green laver chips is shown in Figure 1. First, 20 g of green laver powder, 20 g of frozen surimi, and 60 g of flour, as the major ingredients, were homogenized at 10,000 rpm for 1 min in a homogenizer (T25-S1, IKA Labortechnik, Staufen, Germany). Distilled water (90 mL), in which sugar (12 g), table salt (1 g), roasted soybean powder (1.5 g), and a leavening agent (GDL: NaHCO₃ = 3 g: 2 g) were dissolved, along with 10 mL of RFS, was then added to the mixture and homogenized for 1 min to make the dough. The prepared green laver dough was transferred into a customized acrylic frame (150 mm width × 250 mm length × 15 mm thickness) and spread into a sheet using a rod (40 mm diameter × 385 mm length), then cut and molded into rectangular chips (25 mm width × 40 mm length). The molded chips were dried in an oven (OF-22; Jeio Tech, Daejeon, Republic of Korea) at 50 °C for about 2 h until the moisture content was approximately 13–14%. The moisture content of the green laver chips was determined during processing using a moisture analyzer (Ohaus MB 25, Pine Brook, NJ, USA). The dried green laver chips were heated in an air fryer (MC35A8599LE; Samsung Co, Port Klang, Malaysia) at 195 °C for 60 to 80 s to obtain the final product yielding 35.26% (Figure 2). The heating time of the air fryer and the optimum green laver-to-surimi ratio (%) used as the major ingredients were determined using response surface methodology (RSM).

### 2.4. Proximate Composition Analysis

The proximate composition of the raw materials, including green laver surimi, rice flour, and flour was determined based on the AOAC method [31] as follows: the moisture content was measured using the oven-drying method, crude protein was determined by the semi-micro Kjeldahl method, crude fat was extracted using the Soxhlet method, and crude ash was quantified through the dry ashing method. The carbohydrate content (%) was calculated as the difference from 100% after summing the moisture, crude protein, crude fat, and crude ash contents.

### 2.5. Texture Profile Analysis

The brittleness and hardness of the green laver chips were measured following the method of Jeong et al. [22], modified by the method of Stamataki et al. [32]. Brittleness (N) was evaluated with a texture analyzer (TA-XT2 Plus, Stable Micro Systems Ltd., Godalming, UK), with a three-point bend rig (Stable Micro Systems Ltd., HDP/3PB, Godalming, UK) as the probe. The measurement conditions were as follows: with a pre-test speed of 5.00 mm/s, a post-test speed of 5.00 mm/s, a force of 100 N, a trigger force of 0.049 N, a probe height of 35 mm, and a probe distance of 15 mm. Hardness was set to 60% strain using a plunger (Stable Micro System P/0.255, Godalming, UK) under the same conditions as the brittleness measurement method, and the height of the first peak was measured as the hardness value (N). The results are presented as the mean ± standard deviation (*n* ≥ 3).

### 2.6. Puffing Ratio Analysis

The puffing ratio was assessed using the seed replacement method with waxy millet [33] and calculated by the following equation:

Puffing ration (%) = (V_2_ − V_1_/M_0_) × 100

where M_0_ is the dry weight (g), V_1_ is the volume before puffing (mL), and V_2_ is the volume after puffing (mL).

### 2.7. Volatile Flavor Analysis

The analysis of the volatile flavor components was conducted following the method outlined by Cha and Wang [26]. Flavor adsorption was performed using a solid-phase microextraction (SPME) device (Supelco, Inc., Bellefonte, PA, USA), with a polydimethylsiloxane/divinylbenzene (PDMS/DVB) fiber (65 μm coating thickness). The fiber was activated at 220 °C (GC injection port) for 30 min before analysis. A 6 g sample, 18 mL of deodorized distilled water, and 1 μL (101.14 ng) of hexyl acetate (internal standard, Sigma Co., St. Louis, MO, USA) were placed in a 20 mL headspace glass vial (Supelco, Inc., Bellefonte, PA, USA) and sealed with an aluminum crimp seal (20 mm, open center) and a polytetrafluoroethylene (PTFE)/silicone septum (60 mils). The sample was stirred at 40 °C for 50 min with a magnetic bar while the fiber was exposed to adsorb volatile flavor compounds in the vial. The volatile flavor components were extracted using the SPME method, with each sample undergoing the process three times.

The volatile flavor components adsorbed by the SPME method were thermally desorbed at the injection port (220 °C) for 10 min using a Perkin Elmer 600T GC/MSD (Perkin Elmer Co., Fremont, CA, USA). An Elite Wax capillary column (60 m × 0.25 mm i.d. × 0.25 μm film thickness, Perkin Elmer Co., Fremont, CA, USA) was used with a He carrier gas at 1.0 cm/s. The oven temperature was initially set to 40 °C and held for 5 min, then increased at a rate of 4 °C/min to reach 220 °C, where it was maintained for 10 min. MSD conditions included a 220 °C interface, 204 °C ion source, 70 eV ionization energy, mass range, 33–350 a.m.u., and 1500 V electron multiplier voltage [26]. Compound identification used the retention index (RI) and the NIST (National Institute of Standards and Technology, version 2.3) standard MS library database (Perkin Elmer Co., Fremont, CA, USA). The relative content (factor = 1 ng/g) of the volatile compounds was calculated using hexyl acetate as the internal standard.

### 2.8. Optimization of Processing Conditions for Green Laver Chips Using RSM

The amount of surimi added and the heating time in the air fryer, which are the factors that most influence the texture strength and quality of the green laver chips, were set as independent variables, and response surface analysis was performed using overall acceptance from sensory evaluation and texture (brittleness) as dependent variables (Figure 1). Specifically, the response surface method (RSM) was developed based on the central composite design (CCD) of the RSM, utilizing Minitab Statistical Software (Version 19, State College, PA, USA). The green laver-to-surimi ratio and heating times in the air fryer were set in the ranges of 20 to 30 g% and 60 to 80 s, respectively, through preliminary experiments, and were encoded into 5 levels (−α, −1, 0, +1, and +α) based on the central point. The experimental design included 4 cube points (±α, 0), 4 axial points (±1), and 6 central points (0). The content of flour was calculated as [100 g − (green laver + surimi content)].

### 2.9. Sensory Evaluation

The sensory evaluation was conducted after obtaining approval (No. 7001066-202403-HR-017) from the Institutional Review Board (IRB) of Changwon National University. A total of 11 participants (6 males and 5 females) took part, consisting of undergraduate and graduate students from Changwon National University (aged over 3rd year), who had previous experience with sensory testing for more than 1 year in laver chip processing, especially those who had experience performing QDA in RFS processing experiments, and who had achieved consistent sensory values between samples. In addition, to develop the MZ generation’s preferred chips, they participated in the experiment through a two-month pre-test on the characteristics, taste, and odor of green laver chips in a mini-group method of consumer testing.

Samples were provided in opaque plastic containers (polystyrene: ø 70 mm) (Aju Tech, Hwaseong, Republic of Korea) assigned a random three-digit code. The sensory evaluation was carried out in independent spaces between panels, with bottled water provided for rinsing the mouth between samples, and a one-minute rest was taken between samples.

The optimal mixing ratio of surimi and green laver, the optimal amount of leavening agents (GDL and NaHCO_3_) as sub-materials, and the optimal amount of roasted soybean powder added to the processing of green laver chips were performed by the 9-point rating method (9 = most preferred, 1 = least preferred) of overall acceptance. On the other hand, the optimal conditions between the free amino acid content added to the RFS preparation and the RFS products prepared from these were determined by the ranking method (1st = 4 points, 2nd = 3 points, 3rd = 2 points, and 4th = 1 point).

### 2.10. Statistical Analysis

All experimental data (*n* ≥ 3) were reported as mean values standard ± deviations. As a preliminary experiment, the physical properties (brittleness, hardness, and puffing ratio) between products with different green laver and surimi mixing ratios, between products according to the amounts of leaven agents added, and between products according to the heating time in the air fryer were significantly verified. On the other hand, the difference in free amino acid content in the processing of RFS, the significant difference verification between green laver chip products with RFS, and the significant difference between products according to the amount of roasted soybean powder added were also verified. Furthermore, the results were evaluated using one-way analysis of variance (ANOVA) with the SPSS statistical software (version 27.0, IBM SPSS Statistics, Chicago, IL, USA). Differences between mean values were determined to be significant at the *p* < 0.05 level using Duncan’s multiple range test.

## 3. Results and Discussion

### 3.1. Preliminary Experiments for the Processing of Green Laver Chips

Table 1 summarizes the proximate composition of the raw materials, including green laver surimi, rice flour, and flour. The moisture content of green laver was 6.01 ± 0.29%, with crude protein at 15.62 ± 0.42%, carbohydrates at 69.48 ± 0.84%, crude fat at 0.71 ± 0.09%, and crude ash at 8.18 ± 0.04%. Surimi exhibited a moisture content of 75.52 ± 0.08%, crude protein of 17.83 ± 1.15%, crude fat of 1.26 ± 0.10%, crude ash of 0.47 ± 0.00%, and carbohydrates of 4.92 ± 1.33%. The crude protein content in rice flour and flour was 6.27 ± 0.09% and 11.00 ± 0.00%, respectively, while the crude fat content was 1.09 ± 0.06% and 1.60 ± 0.00%, respectively.

**Figure 3:**
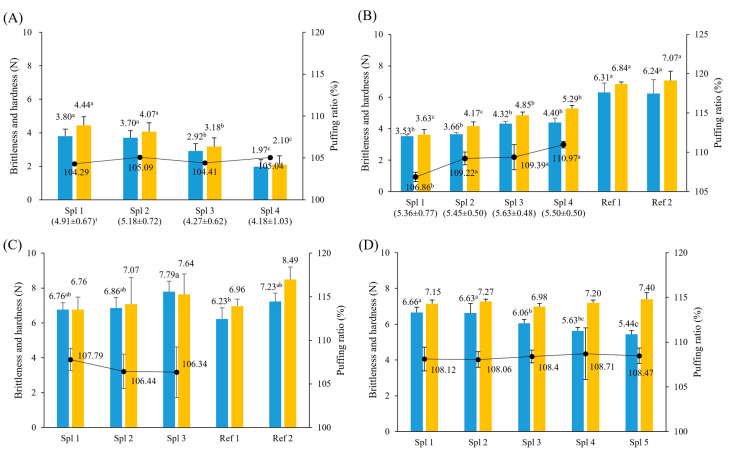
Preliminary experiments for the making of green laver chips. (**A**) Spl (sample) 1 consisted of green laver and surimi at a ratio of 10:10 g%; Spl 2 at 20:20 g%; Spl 3 at 30:30 g%; and Spl 4 at 40:40 g%. Rice flour ratio (g%) was set to 100-(green laver and surimi), and heating time was 90 s. (**B**) Spl 1, NaHCO_3_ 2 g only, and Spl 2, 3, 4 were 2 g of NaHCO_3_ plus with GDL 1 g, 2 g, 3 g, respectively, under the condition of the ratio of green laver (20%): surimi (20%): rice flour (60%), heating time 90 s, and ref (reference, commercial snacks). (**C**) Spl 1, green laver and surimi 20:20 g%; Spl 2, 20:30 g%; Spl 3, 20:40 g%. Flour ratio (g%) was set to 100-(green laver and surimi), and heating time was 60 s, and ref (reference, commercial snacks). (**D**) Spl 1, heating time (HT) 60 s, Spl 2, 3, 4, 5 were 70 s, 80 s, 90 s, and 100 s of HT, respectively, under the condition of a composition ratio of green laver (20%), surimi (20%), and flour (60%). Brittleness (■), Hardness (■), puffing ratio (-●-), and sensory evaluation value (numbers in parentheses). ^a–c^ Means of the same superscript do not differ significantly, as determined by Duncan’s test (*p* < 0.05).

The experiment was conducted under conditions to ensure that the main ingredient, green laver, could be added as much as possible to process green laver chips. Therefore, a four-step preliminary experiment was carried out to establish the amount of green laver powder, frozen hairtail surimi, and rice flour as the major ingredients, leavening agents (NaHCO_3_ and GDL), and the heating time of the air fryer (Figure 3). First, as a result of testing the ratio of the major ingredients (surimi and green laver) (Figure 3A), there was little change in physical strength (brittleness and hardness) within the range of 10:10 g% and 20:20 g%. However, after that, as the content ratio increased, the physical strength tended to decrease significantly due to the increase in the moisture content of the dough (*p* < 0.05). However, the puffing ratio remained almost unchanged within the 104.29–105.09% range in all samples (*p* > 0.05). As the ratio of green laver and surimi increased to more than 20:20 g%, the sensory evaluation showed that green laver’s off-flavor and fishy taste became stronger, decreasing preference. Therefore, the ratio (20:20 g%) with the highest sensory score was selected as the ratio of the major ingredients (60 g% rice flour).

Next, an experiment was conducted to determine the content of the leavening agents (NaHCO_3_ and GDL) as minor ingredients in processing green laver chips (Figure 3B). At this time, the content of NaHCO_3_ was fixed at the same level of 2.0 g based on the sensory evaluation and experimental results of Jeong et al. [22], and the physical strength and sensory evaluation were conducted according to the change in the content of GDL. There was no significant difference in sensory evaluation within the range of 0.0–3.0 g of GDL (*p* > 0.05), but as the GDL content increased, the puffing ratio increased (*p* < 0.05), and both physical strengths (brittleness and hardness) increased, so the GDL content was set to 3.0 g. Compared with the physical strength of brittleness 6.24~6.31 N and hardness 6.84–7.07 N of commercial snacks (Ref 1, 2), the green laver chips in this experiment showed relatively low results in brittleness 3.53–4.40 N and hardness 3.63~5.29 N. This is a gluten-free rice powder characteristic compared to flour and is expected to influence the dough’s physical properties greatly. It was reported that 58.7% of rice powder was appropriate in the processing of laver chips [22], but in this experiment, when the chip was processed by adding green laver and rice powder, it easily crumbled due to the thickness of the chip (1.5 mm), and there were also difficulties in molding. Therefore, to increase the physical strength of the green laver chip, an experiment was conducted to replace it with flour instead of rice powder (Figure 3C).

The puffing ratio of the green laver chips prepared using flour did not show a significant difference (106.34–107.79%) between products (Spl 1~3, Figure 3C), but slightly increased compared to the green laver chips prepared using rice flour (104.29~105.09%). However, in terms of physical strength (brittleness and hardness), compared to rice flour (brittleness 1.97~3.70 N, hardness 2.10~4.07 N), the brittleness increased 2.11~3.43 times compared to rice flour to 6.76–7.79 N, and the hardness increased significantly to 6.76~7.64 N, 1.88~3.22 times. It was also in a similar range to commercial snacks (Ref 1 and 2). Chung et al. [34] reported that when brown rice powder with a higher protein content than rice powder was added when making a snap cookie, the hardness of the cookie was measured as relatively high, which contributes to increasing structural strength as protein and fiber play an important role in the matrix formation of dough. In addition, the hardness of cookies made of flour was higher than that of rice powder because the amylose chain, which has the property of being quickly aggregated by heat, is present in more flour than rice powder. Therefore, flour with a higher gluten content than rice powder improved the chip’s physical properties, and the flour content (60%) that showed physical strength similar to that of commercial snacks (Ref 1 and 2) was finally selected.

Next, a preliminary experiment was performed to determine the optimal conditions for heating time condition in the air fryer during the processing of the green laver chips (Figure 3D). The puffing ratio (108.06~108.71%) and hardness (6.98~7.40 N) according to the heating time (60~100 s) showed little change (*p* > 0.05), but the brittleness gradually decreased as the heating time increased (*p* < 0.05). This result is considered to cause agglomeration and carbonization due to the unbalanced moisture distribution inside the green laver chip as the heating strength (time, temperature, etc.) increases. At the same time, a porous structure is formed, thereby reducing brittleness [35]. The heating conditions in the 60~80 s range were also appropriate for the appearance and texture of the green laver chips, but if it was more than 90 s, the burning phenomenon occurred during the green laver chip manufacturing process.

### 3.2. Determination of Optimum Amounts of Major Ingredients and Heating Time for Processing of Green Laver Chips by RSM

To determine the optimum mixing ratio condition of the green laver chips, an RSM was performed by setting the result values obtained through preliminary experiments (Table 2). As independent variables, the green laver: surimi ratio (g%) and heating time (s) were set, and as dependent variables, brittleness (N) as a measure of physical intensity and overall acceptance (using a 9-point rating method) in the sensory evaluation were determined. Specifically, the green laver: surimi ratio was set in the range of 20~30 g%, and the heating time was set in the range of 60–80 s. A total of 14 random experimental sections were set, with 6 central points (No. 9~14), 4 cube points (±α, 0; No. 1~4), and 4 axial points (±1; No. 5~8) respectively, by encoding (−α, −1, 0, +1, +α) in five stages based on the center point.

The overall acceptance showed the lowest value (6.44 ± 0.55) at No. 1 (X_1_, X_2_: +α, 0) and the highest value (7.45 ± 0.62) at No. 6 (−1, +1). The lowest brittleness (5.94 ± 0.08 N) was also observed at No. 4 (0, −α), while the highest brittleness values (6.36~6.52 N) were seen at the central point. Additionally, as the green laver: surimi ratio increased, overall acceptance decreased, while brittleness increased. On the other hand, as the heating time increased, overall acceptance increased, but brittleness initially increased (until 67 s) and then decreased. The results for the dependent variables according to the independent variables are shown in Table 3. For overall acceptance (Y_1_), the green laver: surimi ratio (X_1_) was significant in the linear model (*p* < 0.05), but not in the quadratic model. On the other hand, the heating time (X_2_) was significant in the quadratic model (*p* < 0.05), but not in the linear model. The R^2^ and lack of fit were 0.8658 and 0.140, respectively. In the case of brittleness (Y_2_), the surimi ratio (X_1_) was significant in the quadratic model (*p* < 0.05), but not in the linear model. The heating time (X_2_), however, was significant in both the linear and quadratic models (*p* < 0.05), with R^2^ and lack of fit values of 0.8293 and 0.088, respectively.

Therefore, the optimum conditions for the green laver chips, based on RSM, were a green laver: surimi ratio of 20:20 g% and a heating time of 80 s. These results were very similar to the ratio of laver and surimi (20:21.3) reported by Jeong et al. [22] in their laver chip processing study. Under these conditions, the predicted overall acceptance and brittleness values were 7.64 and 5.50 N, respectively, while the experimental values obtained were 7.00 ± 0.74 and 5.89 ± 0.59 N, respectively. The absolute residual errors (%) were 8.43% and 7.07%, indicating that the predicted and measured values in the RSM design were highly consistent.

### 3.3. Preparation of Reaction Flavor Solution (RFS) for the Processing of Green Laver Chip Products

**Figure 4:**
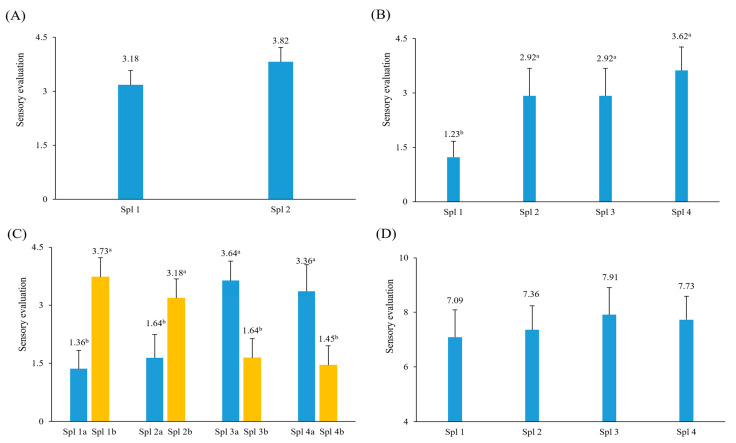
Determination of optimum content of reaction flavor solution (RFS) (**A**–**C**) and roasted soybean powder (RSP) (**D**) for the making of green laver chips. (**A**) Spl 1, three amino acids (AAs) (Gly 0.7 g Pro 0.5 g, and Thr 0.5 g) and glucose 2 g were dissolved in DW 100 mL and then reacted at 98 °C for 90 min; Spl 2, two times the amount of AAs used in Spl 1 except for glucose 2 g. (**B**) Spl 1, AAs (Thr 1.0 g, Pro 1.0 g, and Gly 1.4 g) and glucose 2 g; Spl 2, 1.5 times the amounts of AAs used in Spl 1; Spl 3, the amount of Spl 1 plus Met 0.05 g; Spl 4, the amount of Spl 2 plus Met 0.05 g. (**C**) Spl 1a, same AAs amounts [Spl 1 in (**B**)]; Spl 2a, 3a, and 4a were 1.5, 2.0, and 2.5 times AAs, respectively, under the condition of adding Met 0.05 g. Spl 1b, green laver chip made with RFS of Spl 1A, Spl 2b, 3b, and 4b were green laver chips made with RFS of Spl 2a, 3a, and 4a, respectively. RFS was produced by reacting AAs and glucose (*w*/*v*) within 100 mL of DW at an autoclave (98 °C, 90 min). RFS (■), green laver chips made from RFS (■). (**D**) Spl 1, RSP 0.5 g, Spl 2, 3, and 4 were 1.0 g, 1.5 g, and 2.0 g of RSP, respectively, using a composition ratio of green laver (20 g%), surimi (20 g%), and flour (60 g%), and the ratio of RFS: water was 10: 90 mL, respectively, and then heating time 80 s. ^a, b^ Means of the same superscript do not differ significantly, as determined by Duncan’s test (*p* < 0.05).

In this experiment, to mask the off-flavor of the green laver itself and improve the flavor, free amino acids and monosaccharides were selected as reaction flavor-inducing precursors, and their content ranges were selected through preliminary experiments to generate pyrazines with nutty odors and sulfur-containing compounds with meat-like odors [22,26,28]. The precursors of RFS processed to enhance the flavor of green laver chips were determined through a sensory evaluation (ranking test), with the results shown in Figure 4. First, four kinds of amino acids (threonine, proline, glycine, and methionine) and glucose were selected as precursors for RFS.

Figure 4A shows the sensory evaluation results of RFS (Spl 1) made with three kinds of amino acids (glycine 0.7 g%, proline 0.5 g%, and threonine 0.5 g% *w*/*v*) and glucose (2 g%, *w*/*v*) and Spl 2 made with twice the concentration of Spl 1 (glucose was fixed at 2 g). Although there was no significant difference, Spl 2 was better. Compared to Spl 2, it was considered that the intensity of the odor of Spl 1 was slightly insufficient to mask the off-flavor of green laver chips.

Next, we experimented by adding methionine, which is known to have a good effect in masking the characteristic odor of green laver and transforming it into a meat-like or savory odor [26,28] (Figure 4B). Compared to Spl 1 containing reaction precursors (proline 1.0 g, threonine 1.0 g, glycine 1.4 g, and glucose 2.0 g), Spl 2 (1.5 times the concentration of Spl 1), Spl 3 (concentration of Spl 1 + 0.05 g% methionine), and Spl 4 (concentration of Spl 2 + 0.05 g% methionine) were all significantly better (*p* < 0.05), and among them, Spl 4 was the best.

In Figure 4C, as a result of conducting a sensory evaluation on these RFS (a) and the green laver chips (b) prepared by adding these RFS, Spl 3a containing reaction flavor-inducing precursors (threonine 2.0 g%, proline 2.0 g%, glycine 2.8 g, methionine 0.05 g, and glucose 4 g) was the best, but Spl 1b (threonine 1.0 g, proline 1.0 g, glycine 1.4 g, methionine 0.05 g, and glucose 2 g) was the best when processed as a green laver chip. These results suggested that increasing the concentration of the added reaction flavor-inducing precursors negatively affected the odor and taste of the green laver chips.

Next, roasted soybean powder (RSP) was added based on the results of Spl 1b in Figure 4C to reduce the off-flavor unique to green laver and further enhance the flavor. The sensory evaluation (9-point rating method) according to the concentration of added RSP showed no significant difference (*p* > 0.05) among the concentrations (0.5–2.0 g%), and the nutty flavor and taste increased as the amount added increased. However, the preference for the amount of 1.5 g was evaluated as the highest in sensory preference. It was reported that the addition of RSP (0.5 g%) during the production of brown seaweed (*Undaria pinnatifida*) jam had the effect of offsetting the off-flavor of brown seaweed itself. However, when added excessively, brown seaweed’s fresh odor and taste were lost [36].

### 3.4. Volatile Flavor Components of Green Laver Chips with RFS

**Figure 5:**
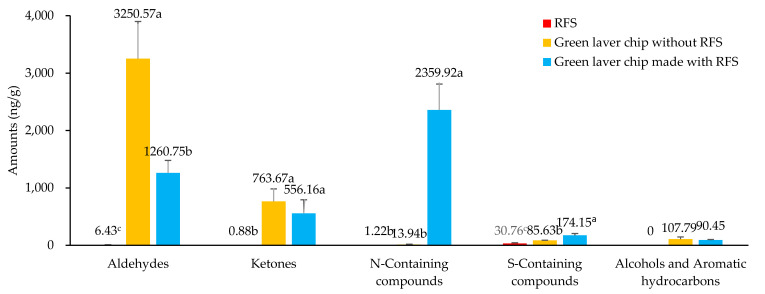
The quantities of categorized volatile compounds identified in the reaction flavor solution (RFS), green laver chip without RFS (GLC), and green laver chip containing RFS (GLCR). ^a–c^ Means of the same superscript do not differ significantly, as determined by Duncan’s test (*p* < 0.05).

The results of analyzing the volatile flavor components of green laver chips (GLC) made without RFS and GLC (GLCR) made with RFS are shown in Table 4 and Figure 5. In total, 58 volatile flavor components were detected and identified as the following groups: 19 aldehydes, 18 N-containing compounds, 13 ketones, 4 S-containing compounds, 2 alcohols, and 2 aromatic hydrocarbons. However, N-containing compounds in RFS were the most numerous with 5 compounds, followed by aldehydes (4), S-containing compounds (3), and ketones (3), while in GLC, there were 16 aldehydes, 8 ketones, and 3 alcohols. These carbonyl compounds accounted for most of the identified compounds (29). On the other hand, in GLCR, N-containing compounds were detected the most, with 15 compounds, followed by aldehydes and ketones, with 5 compounds each.

In particular, among the identified aldehydes, linear C_6_~C_10_ series alkanals, alkenals, and alkadienals were detected only in GLC, except for nonanal. These compounds are known to be produced by lipid oxidation or autoxidation of unsaturated fatty acids [37,38] and are mainly associated with food odors such as green, painty, beany, and rancid [39]. Green laver, which has a high content of ω-3 and ω-6 fatty acids, is believed to react sensitively to lipid oxidation [26].

While only 2-isoamyl-6-methylpyrazine was detected in GLC, 15 N-containing compounds were detected in GLCR, most of which were alkylpyrazines with sweet, corn-like, and nutty odors, and 2-acetylpyrrole with popcorn-like odors were identified [26,40]. Hwang et al. [41] reported that 14 alkylpyrazines were generated in a model experiment with glycine and glucose.

Among the ketones, six straight-chain C_7_~C_10_ and C_15_ series ketones were detected in GLC. These compounds, like aldehydes, are a type of fatty acid oxidation decomposition product that contributes to floral and fruity odors in crustaceans [27]. However, no ketones were detected in GLCR except for 6,10,14-trimethyl-2-pentadecanone. Instead, α- and β-ionone compounds (violet odor), known to be thermally decomposed from carotenoid pigments [42], and geranylacetone, a monoterpene substance (rose, floral odor) [43], were detected.

In S-containing compounds, two straight-chain alkyl sulfides (dimethyl disulfide and dimethyl trisulfide), with soy sauce, fresh garlic, and cooked cabbage-like odors, and a heterocyclic compound, benzothiazole (nutty, popcorn odor), were detected only in RFS [40]. These alkyl sulfides are thought to be generated from methionine, a reaction flavor-inducing precursor, via methional, and are believed to be key flavor compounds in green laver chips because their thresholds are very low at 12 ng/g and 0.01 ng/g, respectively [40]. In a model experiment with methionine and glucose, Yu and Ho [44] reported that methionine alone produced methional, but many pyrazines were produced when reacted with glucose. Based on the results above, it is thought that the alkylpyrazines produced in large amounts in GLCR were formed when the reaction flavor compounds of RFS interacted with the aldehydes abundant in GLC during the heating process with the air fryer, ultimately reducing aldehydes and producing relatively large amounts of pyrazines. It is also believed that these compounds play a significant role in the flavor of GLCR.

## 4. Conclusions

The optimal composition for green laver chips was 20 g% (*w*/*w*) green laver, 20 g% (*w*/*w*) hairtail surimi, and 60 g% (*w*/*w*) flour, with distilled water (90 mL) containing 3 g of GDL, 2 g of NaHCO₃, 1 g of salt, 12 g of sugar, 1.5 g of roasted soybean powder, and 10 mL of reaction flavor solution (RFS). The dough was kneaded, shaped (25 mm width × 40 mm length × 1.5 mm thickness), dried at 50 °C for 2 h, and heated in an air fryer at 195 °C for 80 s. The overall acceptance and brittleness experimental results were 7.00 ± 0.74 and 5.89 ± 0.59 N, respectively, with absolute residual errors of 8.43% and 7.07%. The RFS used in processing contained threonine (1.0 g% *w*/*v*), proline (1.0 g% *w*/*v*), glycine (1.4 g% *w*/*v*), methionine (0.05 g% *w*/*v*), and glucose (2 g% *w*/*v*), which effectively reduced the characteristic green and fishy odors of green laver, producing desirable corn-like, nutty, and popcorn-like aromas through the generation of alkylpyrazines and 2-acetylpyrrole. The analysis revealed that the reaction flavor compounds in RFS interacted with existing aldehydes in green laver chips (GLC) during air-frying, reducing aldehydes and increasing pyrazine production, significantly influencing the flavor profile of GLCR. These findings underscore the effectiveness of combining reaction flavor and air-frying technologies for improving the sensory quality of green laver chips. Further research into the detailed mechanisms of flavor generation could provide deeper insights for broader applications in food processing.

## Figures and Tables

**Table 1:** Summary of the proximate composition of green laver, surimi, rice flour, and flour (g/100 g).

	Green Laver	Surimi	Rice Flour	Flour
Moisture	6.01 ± 0.29	75.52 ± 0.08	7.21 ± 0.15	9.60 ± 0.34
Carbohydrate ^1^	69.48 ± 0.84	4.92 ± 1.33	85.06 ± 0.36	73.00 ± 0.35
Crude protein	15.62 ± 0.42	17.83 ± 1.15	6.27 ± 0.09	11.00 ± 0.00
Crude fat	0.71 ± 0.09	1.26 ± 0.10	1.09 ± 0.06	1.60 ± 0.00
Crude ash	8.18 ± 0.04	0.47 ± 0.00	0.37 ± 0.06	4.80 ± 0.01

^1^ Carbohydrate = 100 − (moisture + crude protein + crude fat + crude ash).

**Table 2:** Coded and decoded independent variables utilized in the RSM design for the production of green laver chips ^1^.

	Symbol	Independent Variable				
		−α	−1	0	+1	+α
Green laver: Surimi ratio (g%)	X_1_	20:20	22.5:22.5	25:25	27.5:27.5	30:30
Heating time (s)	X_2_	60	65	70	75	80

^1^ The flour ratio (g%) was determined as 100—the combined weight percentage of green laver and surimi, and all additives (NaHCO_3_ 2 g, GDL 3 g, table salt 1 g, sugar 12 g) were dissolved in 100 mL of water for a batter for making green laver chips.

**Table 3:** Regression models derived through multiple linear regression analysis of dependent variables, and experimental value based on the result of RSM for making green laver chips.

Dependent Variables	Regression Model Equation	R^2^	*p*-Value	Lack of Fit
Y_1_^1^	6.9061 − 0.2333X_1_ − 0.0815X_2_^2^	0.8658	0.009	0.140
Y_2_^2^	6.4065 + 0.0829X_2_ − 0.0919X_1_^2^ − 0.0775X_2_^2^	0.8293	0.019	0.088
Predicted and actual values				
Independent variables		Predicted value		
X_1_^3^		20		
X_2_^4^		80		
Dependent variables	Predicted value	Experimental Value	Absolute residual error (%)	
Y_1_	7.64	7.00 ± 0.74 ^5^	8.43%	
Y_2_	5.50	5.89 ± 0.59 ^6^	7.07%	

^1^ Brittleness (N), ^2^ Overall acceptance, ^3^ Green laver: surimi ratio (g%), ^4^ Heating time (s), ^5^ Mean ± S.D. (*n* = 11), ^6^ Mean ± S.D. (*n* = 3).

**Table 4:** Volatile flavor compounds in reaction flavor solution (RFS), green laver chip made without RFS (GLC) and with RFS (GLCR) (ng/g).

Compounds by Classification	RI ^1^	Identification Method	RFS	GLC	GLCR
Aldehydes (19)	-	-	6.43 ± 2.28	3250.57 ± 649.28	1260.75 ± 217.98
3-Methylbutanal	900	RI, MS ^2^	- ^3^	655.18 ± 63.84 ^4^	833.81 ± 84.29
Hexanal	1064	RI, MS	-	447.72 ± 133.69	-
Heptanal	1169	RI, MS	-	66.60 ± 0.00	-
Octanal	1279	RI, MS	0.91 ± 0.38	32.29 ± 1.87	-
(E)-2-Heptenal	1305	RI, MS	-	286.23 ± 88.31	-
(E)-5-Methyl-2-isopropyl-2-hexenal	1346	MS	-	-	0.58 ± 0.10
Nonanal	1381	RI, MS	4.49 ± 1.65	131.28 ± 28.10	68.22 ± 15.92
(E)-2-Octenal	1410	RI, MS	-	117.13 ± 30.61	-
*α*-Cyclocitral	1418	RI, MS	-	42.25 ± 23.17	-
(E,E)-2,4-Heptadienal	1471	RI, MS	-	243.08 ± 23.64	-
Benzaldehyde	1498	RI, MS	-	730.20 ± 112.15	-
(E)-2-Nonenal	1513	RI, MS	-	73.66 ± 26.61	-
5-Methylfurfural	1544	RI, MS	0.21 ± 0.02	-	-
(E,Z)-2,6-Nonadienal	1565	RI, MS	-	50.70 ± 16.48	-
Safranal	1630	RI, MS	-	266.97 ± 75.80	347.63 ± 114.01
(E,E)-2,4-Nonadienal	1677	RI, MS	-	52.55 ± 13.79	-
4-Ethylbenzaldehyde	1682	RI, MS	-	50.85 ± 9.93	-
2,4-Dimethylbenzaldehyde	1781	MS	0.82 ± 0.23	-	-
5-Methyl-2-phenyl-2-hexenal	2054	MS	-	3.88 ± 1.29	10.51 ± 3.66
Ketones (13)	-	-	0.88 ± 0.13	763.67 ± 220.32	556.16 ± 237.84
2-Heptanone	1167	RI, MS	-	194.81 ± 4.77	-
6-Methyl-3-heptanone	1236	RI, MS	-	45.40 ± 21.50	-
Hydroxyacetone	1277	RI, MS	0.09 ± 0.00	-	-
2,2,6-Trimethylcyclohexanone	1300	MS	-	54.25 ± 16.04	33.19 ± 16.49
6-Methyl-5-hepten-2-one	1322	RI, MS	0.27 ± 0.06	100.04 ± 36.45	-
2-Decanone	1474	RI, MS	-	137.65 ± 56.04	-
Octa-3,5-dien-2-one	1548	RI, MS	-	160.96 ± 45.65	-
6,10-Dimethyl-5,9-undecadien-2-one	1831	MS	0.52 ± 0.07	-	-
*α*-lonone	1836	RI, MS	-	-	114.44 ± 44.58
Geranylacetone	1841	RI, MS	-	-	42.56 ± 14.19
*β*-lonone	1923	RI, MS	-	-	346.14 ± 149.95
5,6-Epoxy-*β*-ionone	1968	MS	-	62.07 ± 39.29	-
6,10,14-Trimethyl-2-pentadecanone	2115	RI, MS	-	8.49 ± 0.58	19.83 ± 12.63
N-Containing compounds (18)	-	-	1.22 ± 0.32	13.94 ± 6.89	2359.92 ± 450.77
2-Methylpyrazine	1251	RI, MS	-	-	76.79 ± 3.86
2,5-Dimethylpyrazine	1307	RI, MS	0.09 ± 0.05	-	153.92 ± 32.85
2,6-Dimethylpyrazine	1313	RI, MS	0.07 ± 0.05	-	81.87 ± 29.58
Ethylpyrazine	1320	RI, MS	-	-	72.96 ± 21.68
2-Ethyl-6-methylpyrazine	1371	RI, MS	-	-	175.45 ± 23.18
2-Ethyl-5-methylpyrazine	1377	RI, MS	-	-	96.01 ± 11.52
Trimethylpyrazine	1389	RI, MS	0.32 ± 0.07	-	542.33 ± 84.72
Ethenylpyrazine	1413	RI, MS	0.31 ± 0.05	-	
2-Ethyl-3,6-dimethylpyrazine	1431	RI, MS	-	-	342.89 ± 48.19
2-Ethyl-3,5-dimethylpyrzine	1447	RI, MS	-	-	204.87 ± 10.85
3,5-Diethyl-2-methylpyrazine	1479	RI, MS	-	-	169.61 ± 43.36
2-Acetylpyridine	1585	MS	-	-	59.00 ± 3.53
2-Isoamyl-6-methylpyrazine	1604	RI, MS	-	13.94 ± 6.89	-
2-Butyl-3-methylpyrazine	1615	RI, MS	-	-	125.37 ± 33.17
2,5-Dimethyl-3-methylbutylpyrazine	1645	MS	-	-	191.29 ± 85.96
3-Isopentyl-2,5-dimethylpyrazine	1651	MS	-	-	24.84 ± 10.55
2-Acetylpyrrole	1945	RI, MS	-	-	42.72 ± 7.77
2-Ethyl-3,5-dimethylpyridine	2043	MS	0.43 ± 0.10	-	-
S-Containing compounds (4)	-	-	30.76 ± 14.47	85.63 ± 4.01	174.15 ± 31.00
Dimethyl sulfide	759	RI, MS	-	-	174.15 ± 31.00
Dimethyl disulfide	1053	RI, MS	30.06 ± 14.32	85.63 ± 4.01	-
Dimethyl trisulfide	1357	RI, MS	0.51 ± 0.08	-	-
Benzothiazole	1922	RI, MS	0.19 ± 0.07	-	-
Alcohols and Aromatic hydrocarbons (4)	-	-	-	107.79 ± 39.66	90.45 ± 12.45
*m*-Xylene	1117	RI, MS	-	-	36.32 ± 0.46
1-Octen-3-ol	1437	RI, MS	-	10.58 ± 6.58	54.13 ± 11.99
Octanol	1539	RI, MS	-	68.10 ± 15.16	-
1,1,6-Trimethyl-1,2-dihydronaphthalene	1732	MS	-	29.11 ± 17.92	-

^1^ Retention index (RI) of compound on Elite Wax capillary column. ^2^ Compound was provisionally identified based on its mass spectrum (MS) (NIST data) only. ^3^ Not detected. ^4^ Mean value ± S.D. (*n* = 3).

## Data Availability

The original contributions presented in the study are included in the article, further inquiries can be directed to the corresponding author.
